# Determinants of Nurses' Attitudes toward the Care of Patients with Alcohol Problems

**DOI:** 10.5402/2011/821514

**Published:** 2011-05-11

**Authors:** Cara Elizabeth Crothers, Jillian Dorrian

**Affiliations:** School of Psychology, Social Work and Social Policy, University of South Australia, GPO Box 2471, Adelaide, SA 5001, Australia

## Abstract

Nurses (*n* = 49, age = 39 ± 11 y) from an Australian metropolitan hospital completed the Marcus Alcoholism, Seaman Mannello Nurses' Attitudes toward Alcoholism, and the shortened Alcohol and Alcohol Problems Perception Questionnaires. The majority had personal (73%) and/or professional (93%) experience with people with alcohol problems. Not one reported receiving drug and alcohol training. On average, nurses held neutral to positive attitudes toward alcohol problems; however, 14.3% completely disagreed with the statement “I want to work with drinkers,” and 12.5% completely disagreed that they were likely to find working with people with alcohol problems rewarding. Attitudes to care were significantly influenced by age, personal drinking habits, and beliefs about whether patients can be helped, whether alcoholism is a character defect, and the relationship between alcoholism and social status. Negative attitudes towards patient care persist and are influenced by age, personal drinking habits, and beliefs about alcoholism. Specific training in this area may be beneficial.

## 1. Introduction

In one year alone, in Australia, an estimated 81 000 people are hospitalised due to excessive alcohol consumption [1]. The result of this is that an increasing number of nurses are having contact with patients suffering from conditions caused by, related to, or comorbid with alcohol problems. These problems consist not just of issues related to alcohol *abuse* and *dependence *(DSM-IV, 1994, American Psychiatric Association) but also of more generalised issues of misuse, such as binge drinking [1]. Caring for these patients is progressively becoming part of the job, not only of nurses in emergency and specialist drug and alcohol departments, but also of those working on more generalised inpatient wards [2–4]. Around 41% of nurses spend 80–100% of their time responding to alcohol and other drug issues, and the provision of care to these patients can often be a difficult task (Roche and Pidd in [5]). Patients with alcohol problems often provoke a complex array of negative attitudes toward and erroneous stereotypes of “the alcoholic.” These attitudes and stereotypes are present in many societies, including Australia, and nurses, given they arguably spend the most time with patients, must deal with these when treating patients with alcohol problems [6, 7]. In addition, they must also contend with attitudes and stigmas considered unique to the hospital environment, including those depicting patients with alcohol problems as unpleasant, difficult, and unworthy of care [8, 9]. 

These attitudes, beliefs, and opinions, present in the hospital environment, are complex [7, 10, 11]. They include not only the attitudes of society, which depict people with alcohol problems as a dangerous, weak-willed, immoral “drunk” [12, 13], but also stereotypes of these patients as difficult and unrewarding, as well as nurses' own personal or professional experience and their judgements of how deserving these patients are of their care [8, 14, 15]. These beliefs can all shape and influence the relationship that forms between a nurse and his or her patient [15]. 

Negative attitudes have been found in a significant number of nursing populations since the 1960s and 1970s, and although the proportion of nurses with pessimistic attitudes appeared to lessen throughout later decades, negative attitudes still exist today [16, 17]. One of the earlier studies by Wallston et al. [18] found nurses reacted to a hypothetical, male patient, described to participants as an “alcoholic” with various medical conditions, far more negatively than they did the same hypothetical patient, with same medical conditions, when he was not described as an “alcoholic.” When the patient was described as an “alcoholic,” he was perceived as being more unsociable, boring, uncooperative, and unpleasant. Although researchers such as Howard and Chung [16, 17] claim that attitudes appeared to have improved since the 1970s, negative attitudes identified in numerous studies throughout the 1980s and 1990s suggest that attitudes may not have actually changed all that much from those found decades ago [19–22]. At the very least, these studies demonstrate that few steadfast conclusions regarding the nature of nurses' attitudes can be derived from this period of research. 

Likely to be one of the most important studies in the area of nurses' attitudes toward patients with alcohol problems, Allen's [8] study sought to describe and define nurses' attitudes toward this patient population. Significant because of the lack of research since the 1990's and the study's contradictory results, Allen [8] unexpectedly found that nurses had positive attitudes toward patients with alcohol problems. The attitudes of this sample, however, drawn from a community hospital with an inpatient drug and alcohol program, whose staff had been quite active within other hospital wards, starkly contrasted with those of a second sample, drawn from a larger hospital with no treatment program. Nurses from this hospital had far more negative attitudes toward these patients, expressing feelings of disgust, anger, and of being used and having little patience when caring for them. 

Whilst Allen's [8] findings demonstrate the possible effect nurses' education and the support provided by inpatient drug and alcohol treatment programs can have on their attitudes, other factors such as the ward on which they work [23], as well as their own personal and professional experiences [14, 24], can all influence the attitudes and beliefs they have about patients with alcohol problems. Having few experiences with the successful treatment of patients with alcohol-related issues or the existence of alcohol issues within their own families, may negatively impact on a nurse's attitude toward their patient [14] (Maher-Brisen in [25]). 

The existing literature supports a link between nurse attitudes and the care they provide their patients [15, 24, 26]. Indeed, these “cornerstones” ([27], page 9) of patient care can affect not only the amount but also the quality of care that nurses provide [24]. Plumlee, an author in the field of nursing education, states that an “accepting, nonjudgemental, caring” (in [15], page 117) attitude is essential when treating patients with alcohol problems, yet, moralistic, stereotypic, pessimistic, and ultimately counterproductive attitudes still endure [14]. While there has been some research into the disparity between attitudes ideally held by nurses and those that are actually present, this research has focussed primarily on identifying shortfalls in nurses' education in the field of drug and alcohol nursing [28–30]. 

To date, research has largely ignored other factors which may impact on nurses' intentions to engage with a patient with alcohol problems. Yet current efforts in the development of the drug and alcohol workforce recognise that education and training are only one aspect of a wide range of factors that can influence nurses' ability to care for patients with alcohol problems [5]. Without investigations into the influence of individual factors such as nurses' personal characteristics, their attitudes toward alcohol, their beliefs about the causes and symptoms of alcohol abuse problems, and their beliefs about their role in the care of such a patient, our understanding of the nurse-patient dynamic is limited. In light of the amount of contact nurses have with patients experiencing alcohol problems and the influence of their attitudes on patient care, a thorough understanding is vital. This, combined with the lack of Australian research, highlights the need for current work in this area. This study aims to examine personal characteristics, attitudes to alcoholism, and attitudes to care of patients with alcohol problems in a cohort of Australian metropolitan hospital nurses.

## 2. Method

### 2.1. Sample

A survey was conducted at a large Australian metropolitan teaching hospital. Approval for the study was obtained from the University of South Australia Human Research Ethics Committee and the ethics committee at the participating hospital. In order to take part in the study, participants had to be currently employed, enrolled, or registered nurses. Participant recruitment took place on four wards selected for the study based on favourable responses from their Clinical Services Coordinators (2 general medical, 1 thoracic, and 1 cardiology) and in an educational seminar held weekly for any interested nurses from all wards within the general medicine service. The response to the survey (*n* = 51) was 34% of the possible population (a total of 150 questionnaires were handed out or accessible on the wards).

### 2.2. Data Collection

The study was introduced to nurses during an educational session on each of the wards, and questionnaires were then given to the nurses in attendance and others left in staff break rooms for those who did not attend. Questionnaires were given to all of the nurses in attendance at the weekly educational seminar for staff of the general medicine service. Nurses were encouraged to give copies of the questionnaire to fellow nursing staff not working on the wards selected for recruitment. The completed questionnaires could be dropped into a box located in the break rooms of the wards or mailed to the researcher using the postage paid, addressed envelope attached to the questionnaire. Two weeks later, staff on the four wards selected for recruitment were reminded of the study by their Clinical Services Coordinator and via flyers posted in high traffic, easily visible areas (i.e., break rooms and toilets). One week after, the boxes of completed questionnaires were collected and reminders removed.

### 2.3. Measures

An anonymous questionnaire was developed for the study, combining three existing questionnaires with 12 sociodemographic questions. The questionnaire was designed to assess various personal characteristics of the participants, their attitudes towards alcoholics and alcoholism, and their attitude toward the care of patients with alcohol problems. The measures in the 92-question, twelve-page questionnaire are outlined below.

### 2.4. Personal Characteristics

Sociodemographic characteristics were measured using twelve questions based on an Australian study of the determinants of nurses' therapeutic attitude to patients who use illicit drugs [31]. Questions were designed to assess participants' sex, age, role (enrolled or registered nurse), if this was a management role, years of employment, ward on which they were currently employed, education in drug and alcohol nursing, religious affiliation and service attendance, personal use of alcohol, experience with patients with alcohol problems, and personal experience with people with alcohol-related problems.

### 2.5. Attitudes towards Alcoholics and Alcoholism

Nurses' attitudes toward alcoholics and alcoholism were assessed using the Marcus Alcoholism Questionnaire [32] and the Seaman-Mannello Nurses' Attitudes toward Alcoholism Scale [33]. The Marcus Alcoholism Questionnaire is designed to measure attitudes towards “alcoholics” and consists of 40 statements based on the 9 factors shown in Table 1. Items are responded to on a Likert scale that ranges from 1 (completely disagree) to 7 (completely agree). A high score on factors 1, 2, 4, and 9 indicates a positive attitude, and a high score on factors 3, 5, 6, 7, and 8 indicates a negative attitude. The authors of this scale tested it on several sample populations, including health care professionals and experts in the field of alcoholism. Based on this testing, the scale has established reliability of  .90 and established validity (Addictions Research Foundation, in [8]). It has also been used in numerous studies. Martinez and Murphy-Parker [34] included it in their study as a measure of nursing students' beliefs about people with addictions, and a commonly cited study by Allen [8] used the Marcus Alcoholism Questionnaire to examine the attitudes of registered nurses toward alcoholic patients in a general hospital population. 

Two examples of the items on this scale are

1. the alcoholic is a morally weak person,
2. alcoholism is best described as a habit rather than an illness.

The Seaman-Mannello Nurses' Attitudes toward Alcohol and Alcoholism Scale consists of 30 items and also uses a Likert scale for responding to questions which ranges from 1 (strongly disagree) to 5 (strongly agree). However, in the interest of consistency, the 7-point Likert scale used for The Marcus Alcoholism Questionnaire was also used for this scale in the final questionnaire. Items on this scale are divided into 5 subscales shown in Table 1. Although reported in other studies, no reliability or validity psychometrics on the subscales are available [35]. This instrument has been used to examine the attitudes of nurses' at the University Hospital in San Antonio, Texas towards alcoholic patients and is a commonly used tool in Brazilian studies [35, 36]. Many of these studies have found that negative attitudes towards “alcoholic” patients were predominant in nurses working with this patient group [37, 38]. Two examples of the items on this scale are

1. alcoholics deserve hospital space just like any other patient,
2. alcoholics want to stop drinking.

### 2.6. Attitudes toward the Care of Patients with Problematic Alcohol Use

Nurses' attitudes toward the care of patients with problematic alcohol use were assessed using the shortened version of the Alcohol and Alcohol Problems Perception Questionnaire (SAAPPQ) [39, 40]. The SAAPPQ is a validated scale based on factor analysis of the original alcohol and alcohol problems perception questionnaire developed and validated by Cartwright [41]. The SAAPPQ has been used in previous studies as a measure of the attitudes of medical and nursing staff to alcohol assessment and intervention and of GP's attitudes towards working with drinkers [42, 43]. The SAAPPQ has a high degree of correlation with the full length AAPPQ, which has good test-retest reliability and Cronbach's alpha in the range of 0.7 and 0.9 [39, 41]. The validity of the SAAPPQ was demonstrated by Anderson and Clement [39] by comparing both the short and full length scales with other responses from Cartwright's original study. The SAAPPQ consists of 10 items, to which respondents answer using a 7-point Likert scale that ranges from 1 (strongly disagree) to 7 (strongly agree). Items are designed to measure 5 areas as shown in Table 1. Two examples of items on this scale are.

1. I want to work with drinkers,
2. in general, it is rewarding to work with drinkers.

On this scale, a high score indicates a more positive attitude and a low score a more negative attitude.

### 2.7. Statistical Analyses

Data were analysed using version 17 of the Statistical Package for the Social Sciences (SPSS version 17.0). Results are reported in four steps. First, an overview of the study sample is presented. Second, the results of an assessment of the reliability and factor structure of the Marcus Alcoholism Questionnaire, the Seaman Mannello Nurses' Attitudes toward Alcoholism Scale, and the Shortened Alcohol and Alcohol Problems Perception Questionnaire are reported. Third, the average attitude found among the sample is reported. Fourth, exploratory univariate analyses examining the association between nurses' attitudes towards alcoholics and alcoholism and nurses' personal characteristics are reported. Finally, multiple regression analysis is presented, assessing the relationship between nurses' attitudes towards “alcoholics” and alcoholism and their attitudes toward the care of patients with alcohol problems. A separate model was tested for each of the SAAPPQ subscales. In the first step in the regression analyses, all the variables of interest were entered into the model simultaneously, and significant variables were noted. Nonsignificant variables were then removed from the analysis and the model rerun, resulting in a preliminary main effects model [44]. Each nonsignificant variable was then entered back into the model one at a time in order to test if it was significant or if it substantially changed the parameter estimates of the other variables. If any of the individual predictors appeared important at this stage, they were added to the preliminary main effects model and the analysis was rerun, resulting in a main effects model. The final model, therefore, contained only significant individual variables and/or variables that were important because of shared variance with other variables [44].

## 3. Results

### 3.1. Overview of the Study Sample

One hundred and fifty questionnaires were distributed, of which 49 (33%) were returned complete and 2 were returned with less than 70% of the questions completed. The two incomplete questionnaires were removed from the data set. The study sample was predominately female (92%) with a mean age of 39 years (SD: 11 years, range 21–63 years). Sixty-five percent of the sample identified as having a religious denomination, and, of those, twenty six percent never attended religious services, thirty two percent attended services once or twice a year, and the remaining forty two percent attended services more than twice a year. Just under half the sample reported never using alcohol, or doing so only on special occasions (42%), while twenty-five percent reported using alcohol once a week and thirty-three percent more than once a week. More than half the sample reported consuming between one and two standard drinks on any one occasion (57%), as opposed to those who did not consume any (16%) or who consumed more than two standard drinks (27%). In terms of their nursing role, sixty-nine percent of the participants were registered nurses, and both registered and enrolled nurses generally worked on general medical (37.5%), surgical (25%), or other type of ward (37.5%). The majority of the sample had personal (73%) and professional (94%) experience with people with alcohol problems.

As only four participants were male, sex was not used as a variable in analyses. Variables relating to participants' positions in management (*n* = 4) and professional experience with patients with alcohol-related problems (*n* = 46) were also not used in analyses as there were not enough participants or, in the case of professional experience, too many participants, to warrant using these variables. Years of employment was discarded as a variable as it was positively skewed and, after being transformed, was highly correlated with age. Drug and alcohol training also could not be used as a variable in analyses because not a single participant reported ever receiving any training in this area.

### 3.2. Reliability Analysis

Table 2, column 3, shows Cronbach's alpha coefficient for each of the subscales of the Marcus Alcoholism Questionnaire (Marcus), the Seaman-Mannello Nurses' Attitudes toward Alcohol and Alcoholism Scale (Seaman-Mannello), and the Shortened Alcohol and Alcohol Problems Perception Questionnaire (SAAPPQ). Items 3, 4, and 6 of the Marcus questionnaire were reverse scored. The table shows that only subscales 5, 6, and 7 on the Marcus questionnaire and 2, 3, 4, and 5 on the SAAPPQ meet the value of  .7, the acceptable value for Cronbach's alpha [45]. Following the deletion of items that correlated less than  .4 with the other items on each of the subscales, the reliability analysis was rerun. The process of discarding poorly correlated items and rerunning the reliability analysis was followed until Cronbach's alpha could no longer be increased. 

To confirm the factor structure of the subscales to be used in analyses, a principal axis factor analysis was carried out for the Marcus, Seaman-Mannello, and SAAPPQ scales. Only the subscales with Cronbach's alpha scores of 0.7 (or very close to 0.7) were included in the factor analysis. Oblique rotation was used (Promax with Kaiser Normalisation). Factors were forced in each analysis according to the number of subscales entered. Items which did not load onto their corresponding factor were discarded, and the factor analysis was rerun. Reliability analyses were then run on the resulting subscales. On the basis of the strength of Cronbach's alpha 4 subscales of the Marcus questionnaire (recovery potential, character defect, social status, and illness) and 3 subscales of the Seaman-Mannello scale (satisfaction, “helpable,” and personal drinking) were used in analyses. Factor 1 (motivation) of the SAAPPQ was split and its two items included separately in analyses. Optimised subscales with Cronbach's alpha scores of, or close to, 0.7 are shown in Table 2, column 4.

### 3.3. Overview of Attitude Subscales

Table 3 presents the means and standard deviations of each of the Marcus, Seaman-Mannello, and SAAPPQ subscales. Mean score limits are 1.00–7.00, with 3.50 being the dividing point between a high factor score and a low factor score. A high score on any of the Marcus questionnaire subscales indicates a negative attitude. While a high score on the SAAPPQ or the Seaman-Mannello subscales indicates a positive attitude, with the exception of the “Pessimism is the most realistic attitude to take toward drinkers” item in which the originally scored responses were used to aid with interpretation and where a low score indicates a positive attitude (i.e., a higher score represents more pessimism). The table shows that the mean factor scores on all of the Marcus subscales (recovery potential, character defect, social status, and illness) are below 3.5, only one of the Seaman-Mannello subscale means was below 3.5 (satisfaction), four mean factor scores for the SAAPPQ were above 3.5 (work satisfaction, role adequacy, role legitimacy, and self-esteem), and 2 were below (desire and pessimism). 

### 3.4. Univariate Analyses

*Marcus Questionnaire*. Between-groups *t*-tests and ANOVAs found no significant group differences (role, personal experience, religious affiliation, service attendance, ward, alcohol consumption, and number of standard drinks) in nurses' attitudes toward alcoholism (*P* > .05). Correlations between age and all of the Marcus subscales were between −.07 and −.27 and were not significant, except for the illness subscale where there was a significant, moderate, negative correlation (*r* = −0.52, *P* < .001) subscales. 

*Seaman-Mannello Scale*. A significant effect of alcohol consumption on scores on satisfaction was found (*F* = 3.516, *P* < .05) such that there was a significant (*P* < .05) difference for those consuming alcohol more than once a week (mean = 4.89, SD = 0.84) compared to those who never consume alcohol or do so only on special occasions (mean = 2.99, SD = 1.15). There was also a significant effect of the number of standard drinks on personal drinking (*F* = 4.853, *P* < .05) such that there was a significant (*P* < .05) difference for those reporting more than 2 standard drinks (mean = 4.71, SD = 0.82) or 1-2 standard drinks (mean = 4.82, SD = 1.16) compared to those reporting no standard drinks (mean = 3.50, SD = 1.07) (*P* < .05). Correlations between age and all of the subscales of the Seaman-Mannello scale were between −.07 and −.27 and were not significant.

*SAAPPQ*. Between-groups *t*-tests and ANOVAs found no significant group differences (*P* > .05). Correlations between age and all of the subscales of the SAAPPQ were between −.01 and  .05, except for the pessimism subscale, which had a significant, moderate, negative relationship with age (*r* = −0.35,  *P* < .05).

### 3.5. Multivariate Analyses

A series of multiple regressions was used in order to assess whether there was a positive relationship between nurses' attitudes toward alcoholism and their attitudes toward the care of patients with alcohol problems. All of the Marcus and Seaman-Mannello subscales established as reliable above were entered into the multiple regression analyses. Age, alcohol consumption, and number of standard drinks variables (shown to be related to some of the Marcus and Seaman-Mannello subscales in the univariate analyses) were also entered into analyses. The final models are shown in Table 4. This table shows that, for the SAAPPQ subscale work satisfaction, scores on Seaman-Mannello satisfaction subscale had a significant, positive effect and accounted for 41.6% of the variance. 

Table 4 also shows the results of the multiple regression for self-esteem. These results show that although consuming alcohol once a week had a significant positive effect on participants' self-esteem, accounting for 18.4% of the variance in scores on the self-esteem subscale, the addition of the recovery potential subscale to the model shows that recovery potential also had a significant, yet negative effect on participants self-esteem. Together, consuming alcohol once a week and scores on the recovery potential subscale accounted for 45.4% of the variance in scores on the self-esteem subscale.

Multiple regression analyses of the desire subscale, revealed that, when controlling for social status, consuming alcohol more than once a week and scores on the Seaman-Mannello satisfaction subscale had a significant, positive effect on participants' desire to work with drinkers. Together, these variables account for 38% of the variance in participants' scores on the desire subscale. 

Lastly, Table 4 shows the outcome of the multiple regression analyses performed on the pessimism subscale. The table shows that, while consuming more than 2 standard drinks had a significant, positive effect on scores on the pessimism subscale, accounting for 11.5% of the variance in scores on this subscale, the addition of the character defect subscale and age variable to the model had a significant effect. Analyses also showed that, when controlling for age, the character defect subscale, which had a significant, positive effect, and the consuming more than 2 standard drinks variable accounted for 34% of the variance in participants scores on the pessimism subscale. 

Multiple regression analyses of the SAAPPQ subscales role adequacy and role legitimacy revealed that there were no variables which had a significant effect on either outcome variables.

## 4. Discussion

### 4.1. Summary of Findings

This study sought to explore the nature of nurses' attitudes toward patients with alcohol *abuse* and *dependency* problems and to investigate the relationship between these attitudes and nurses' attitudes toward the care of such patients. Findings indicate that, contrary to expectations, on average nurses have rather positive, or at least quite neutral, attitudes towards patients with alcohol problems. Despite this, 14.3% responded that they completely disagreed with the statement “I want to work with drinkers,” and 12.5% indicated they completely disagreed that they were likely to find working with alcoholics rewarding. 

Also identified in the study was an association between nurses' personal characteristics and attitudes toward alcohol problems. Older nurses believed more strongly that alcoholism is an illness and that pessimism is not the most realistic attitude to take toward drinkers. An association between the average number of times per week a nurse drinks and their personal and professional satisfaction in working with patients with alcohol problems was evident, as well as an association between the number of standard drinks they usually consume and nurses personal attitudes toward drinking. 

A positive relationship between some, but not all, aspects of nurses' attitudes toward alcohol problems and their attitudes toward the care of patients with such problems was also found in the current study. Moreover, both positive and negative relationships were found between various aspects of nurses' attitudes toward the care of patients with alcohol problems and nurses' age and personal drinking habits. These results indicate that at least some of the attitudinal aspects measured by the Marcus and Seaman-Mannello scales, as well as a nurses' age and drinking habits, are important predictors of their attitude toward working with patients with alcohol problems.

### 4.2. Nurses' Negative Attitudes

Although the generally negative attitudes towards patients with alcohol problems observed in previous research were not found in the current study, around 12% of the participants had strong, negative feelings toward working with such patients. Overall, nurses instead appeared to have, on average, attitudes that were consistently quite positive, if not neutral. This suggests that there may have been a shift in attitudes since the previous research in the 1980's and 1990's. One possible reason for this shift may be a growing acceptance of people with alcohol problems, likely related to the increase in our knowledge about the physiological, behavioural, and cognitive causes of these conditions [46]. It may also be that the now nearly universal acceptance of alcohol abuse and dependence as a disease, and not a moral choice, has encouraged a greater acceptance of alcohol problems [16, 17].

### 4.3. Association between Personal Characteristics and Attitudes

Previous research has suggested that nurses' personal characteristics, such as education, religion, and personal experiences, influence their attitudes towards alcohol problems [8, 16, 17]. The results of this study build on that suggestion, showing that age also influences these attitudes. A review of the available literature though showed that, in spite of the recognition of the influence of personal characteristics, no studies to date had examined the possible effects nurses' own personal use of alcohol might have on their attitudes toward alcohol problems. Interestingly, just under half of the participants in this study reported never using alcohol or doing so only on special occasions (42%), which is higher than the national average (33.5% of Australians drink less than weekly, and 9.3% never drink a full serve of alcohol) [2, 3].

Results show that nurses' personal drinking habits had an effect on their attitudes toward alcohol problems. Nurses who drink, on average, more than once a week were shown to be less likely to find working with patients with alcohol problems rewarding and questioned their ability to successfully deal with such a person, as well as feeling discomfort or embarrassment when dealing with their alcohol problems. In addition, the number of standard drinks they usually consume also influenced their beliefs about alcohol problems. Nurses who consumed alcohol in any quantity were more likely to believe that the danger is in the alcohol, not in the person, and, interestingly, that the consumption of alcohol in any quantity is harmful. This raises interesting questions about the relationship between nurses' perceived risks of alcohol consumption, their behaviour in spite of that risk, and the attribution of blame when a negative outcome (such as alcohol problems) occurs.

A lack of training in the treatment of alcohol problems was also identified in the study. Despite 73% of the sample having had experience with people with alcohol-related problems in their personal lives and 94% indicating they had experience with patients with such problems, not a single participant had ever received specialist training in the treatment of alcohol issues. This finding is supported by those of a recent Australian study [29, 30] that found around 60% of the nurses in their sample had not received any in-service alcohol issues training. It also supports the suggestion by Arthur [47] that drug and alcohol issues do not appear to receive adequate attention within the nursing curricula, despite significant proportions of hospital patients having alcohol-related illnesses. However, despite the apparent lack of education in alcohol issues within the current study's sample, nurses indicated that they felt they had reasonably adequate knowledge and skills in working with such patients. This may suggest that nurses feel they learn enough “on the job,” or through previous education and personal experiences, to work with patients with alcohol-related problems. An investigation into the amount and adequacy of knowledge that nurses feel they gain through work and personal experiences would therefore be a useful addition to the existing literature.

### 4.4. Relationship between Attitudes and Attitudes toward Care

*Work Satisfaction*. It was found that nurses' ratings of work satisfaction on the Seaman-Mannello Nurses' Attitudes toward Alcohol and Alcoholism Scale were a significant predictor of their score on the Shortened Alcohol and Alcohol Problems Perception Questionnaire's (SAAPPQ) measure of work satisfaction. While this is not necessarily surprising, given the similarity in the concepts measured by the two scales, it does have important implications. Notably, a high score on the SAAPPQ's measure of work satisfaction relates not only to how a nurse feels regarding patients with alcohol problems, such as is measured by the work satisfaction score of the Seaman-Mannello Nurses' Attitudes toward Alcohol and Alcoholism Scale, but also to a nurses' commitment to working with these patients, that is, the degree to which they seek to engage in the treatment of patients with these problems [10]. The strength of this relationship, therefore, indicates that nurses' attitudes regarding how much they like, and feel rewarded by, working with patients with alcohol problems, are an important determinant of the extent to which nurses are actually willing to engage in this work.

*Task-Specific Self-Esteem*. In addition, findings indicate that self-esteem (in working with patients with alcohol problems) is greater in nurses that consume alcohol once a week than in those who never consume alcohol (or who do so only on special occasions) or in those who do so more than once a week. However, as the belief that people with alcohol problems do not, and cannot be helped to, recover increases, self-esteem levels decrease. Shaw and colleagues [48] offer a possible explanation for this relationship found between nurses' beliefs regarding patient recovery and their self-esteem. According to these authors, the patients identified as the most unlikely to improve are the most threatening to a person's professional (task-specific) self-esteem. Moreover, the more unlikely recovery is deemed to be, the less confident the agent treating them will feel in responding. Therefore, the finding that, as the belief that people with alcohol problems cannot recover increases, nurses self-esteem in treating such people decreases, supports existing theory. 

“*I Want to Work with Drinkers.*” Alcohol consumption was also found to be a predictor of nurses' motivation to work with drinkers. This item on the SAAPPQ, treated as a subscale, is an indication of nurses' motivation and willingness to work with patients with alcohol problems [41]. Results show that consuming alcohol more than once a week and higher levels of personal and professional satisfaction in working with people with alcohol problems are significant predictors of a greater desire to work with this patient group. 

“*Pessimism Is the Most Realistic Attitude to Take toward Drinkers.*” Results showed that it is also a combination of factors that predict the degree to which nurses' feel that pessimism is the most realistic attitude to take toward drinkers. Nurses' pessimism, also a measure of nurses' motivation to work with people with alcohol problems [41], increases in those who are younger and who consume, on average, more than two standard drinks on any one occasion, and as their beliefs that people with alcohol problems are weak-willed increase.

### 4.5. Strengths and Limitations of the Study

Although the small sample size [8, 49] and low response rate [50] are not unusual in the area of research, they do, along with mainly female sample, make it difficult to generalise the results to a wider population. Furthermore, the study was conducted in only one hospital, with much of the sample working on similar wards. Greater variety between wards may have resulted in more varied attitudes, as indicated by previous research [23]. Future studies would likely benefit from the inclusion and comparison of very different departments, such as specialist alcohol units, psychiatric units, emergency departments, or wards where alcohol-related issues, such as liver and kidney failure [51], may be prevalent. 

The use of quite strong, emotive language in the questionnaire should also be taken into account. The choice of terms such as “alcoholic” and “care of,” used in the questionnaire, was designed to elicit strong responses, and had terms with fewer negative or implicative connotations been used, different findings may have emerged. Other possible limitations include self-report response bias in the questionnaire; however, given the questionnaire was anonymous and clearly stated to have no bearing on participants' employment, social desirability bias was minimised as much as possible. 

The major limitations of this study relate to the validity and reliability of the scales used in the questionnaire. Of a combined possible fourteen factors contained in the Marcus and Seaman-Mannello Scales, only four had internal consistencies which met the required cut-off [45] and only three with that were close enough to this cut-off to be included in analyses. Furthermore, factor analyses of the items of the two scales showed that several items were inconsistent with the original factor structure created by the authors. Therefore, a reduced number of attitudinal aspects were included in study. These findings contrast greatly with numerous studies, which over the past three decades have used and described these scales as valid and reliable [8, 34–36]. In order to investigate all attitudinal aspects, development and psychometric assessment, in particular for those scales that were not reliable in this sample (emotional difficulties, loss of control, the alcoholic as a steady drinker, harmless voluntary indulgence, addiction liability, case disposition, and perception of personal characteristics of alcoholic persons) would be of great benefit. 

Despite these limitations, this study has provided insight into the current state of nurses' attitudes toward alcohol problems and the care of patients with these problems. It appears to be the most recent study of nurses' attitudes toward alcohol problems (in at least fifteen years) and the first to investigate their attitudes toward caring for patients with these problems. The approach used allowed the establishment of the relative importance of both personal characteristics and attitudes and therefore has important implications for nursing practice and workforce development. A recent Australian study showed that a significant proportion of nurses who are not specialist drug and alcohol workers are spending a considerable amount of time working with patients with these problems [5]. In light of the important role attitudes play in the care patients receive from nurses, identifying areas where these attitudes are particularly negative or where development may be most beneficial has important repercussions for patient care [15]. It also has important implications for nurses themselves. Given that this and other studies show how often nurses encounter patients with alcohol problems, it seems reasonable to conclude that patient outcomes, nurse retention rates, and workplace environments would improve when nurses feel more secure, satisfied, motivated, and generally happier, in their role. The more information those responsible for the development and implementation of nurse education and workplace training have regarding the areas where negative attitudes, such as pessimism regarding the success of treatment, inadequate education, and reduced motivation exist, the better they may develop and improve these areas [5, 50].

### 4.6. Suggestions for Future Research

This study has indicated a relationship between nurses' attitudes toward alcohol problems and attitudes toward the care of patients with these problems. Future research could further develop this relationship and investigate to what extent nurses' attitudes toward care influence their actual care of these patients. Prospective Australian studies could also build on the results of this and other researches by conducting qualitative research into the nature of nurses' attitudes toward this patient group. This type of research has already been conducted in Brazil, and was shown to better capture a wide range of beliefs, including those not contained within the Marcus, Seaman-Mannello, or SAAPPQ scales [36].

## 5. Conclusions

Given that nearly all nurses surveyed had professional experience involving patients with alcohol problems, with no reports of training in this area, this may be an important addition to education programs. On average, the nurses had neutral to positive attitudes regarding alcohol problems, which is encouraging compared to the predominantly negative views uncovered by research from the 1980s and 1990s. Despite this, roughly one in seven participants reported negative attitudes towards the care of patients with alcohol problems. Age and personal drinking habits, as well as beliefs about alcoholism, can influence attitudes towards patient care.

## Figures and Tables

**Table 1:** Factors and their interpretation for The Marcus Alcoholism Questionnaire ([32], pages 3, 4), the Seaman-Mannello Nurses' Attitudes toward Alcohol and Alcoholism Scale ([33], page 166), and the Shortened Alcohol and Alcohol Problems Perception Questionnaire (SAAPPQ) ([39], page 754).

Marcus: high score indicates the belief that…	
(1) Emotional difficulties as causes of alcoholism: “emotional difficulties or psychological problems are an important contributing factor in the development of alcoholism”	
(2) Loss of control: “the alcoholic is unable to control his drinking behaviour”	
(3) Prognosis for recovery: “most alcoholics do not, and cannot be helped to, recover from alcoholism”	
(4) The alcoholic as a steady drinker: “periodic excessive drinkers can be alcoholics” (low score—“person must be a continual excessive drinker in order to be classified as an alcoholic”)	
(5) Alcoholism and character defect: “the alcoholic is a weak-willed person”	
(6) Social status of the alcoholic: “alcoholics come from the lower socioeconomic stratum of society”	
(7) Alcoholism as an illness: “this is not an illness”	
(8) Harmless voluntary indulgence: “the alcoholic is a harmless heavy drinker whose drinking is motivated by his fondness for alcohol”	
(9) Addiction liability: “alcohol is a highly addicting substance”	

Seaman-Mannello: high score indicates the nurse is likely to…	

(1) Case disposition, therapy versus punishment: “believe that alcoholics are physically ill… medical treatment is warranted” (low score—“alcoholics are in good physical health and should be punished for their alcoholism”)	
(2) Personal/professional satisfaction in work with alcoholics: “find working with alcoholics rewarding… enjoy having them as patients and feel comfortable treating them.” (low score—“discomfort and embarrassment when dealing with people with drinking problems”)	
(3) Inclination to identify: ability to help alcoholic patients: “see alcoholics as potentially respectable citizens who can be helped to resume normal lives… alcoholics want to be cured and that the nurse can help them.” (low score—“alcoholics are selfish and do not want to be helped”)	
(4) Perceptions of personal characteristics of alcoholic persons: “see alcoholics as basically unhappy people—lonely, sensitive, doubting their own worth, and having severe emotional difficulties” (low score—“alcoholics as people who are simply excessive drinkers and who do not have psychological problems”)	
(5) Personal attitudes toward drinking: “believe that alcohol per se is not bad. Moderate consumption of alcohol may actually be beneficial.” (low score—“the danger is in the alcohol and not in the person—the consumption of alcohol in any quantity is harmful, if not morally wrong”)	

SAAPPQ: interpretation	

(1) Motivation: “a health professionals motivation or willingness to work with drinkers”	
(2) Work satisfaction: “their expectations of work satisfaction with these clients”	
(3) Role adequacy: “their feelings about the adequacy of their knowledge and skills in working with such patients”	
(4) Role legitimacy: “the extent to which they feel they have the right to work with drinkers”	
(5) Task-specific self-esteem: “their self-esteem in this specific task”	

**Table 2:** Internal consistency of individual subscales in original format and following optimisation.

Scale	Subscale	Cronbach's alpha	
		All subscales	Optimised subscales
Marcus	(1) Emotional difficulties	.553	
	(2) Loss of control	.143	
	(3) Prognosis for recovery	.642	**0.727**
	(4) The alcoholic as a steady drinker	.349	
	(5) Alcoholism and character defect	.836	**0.836**
	(6) Social status of the alcoholic	.730	**0.730**
	(7) Alcoholism as an illness	.709	**0.684** ^a^
	(8) Harmless voluntary indulgence	.355	
	(9) Addiction liability	.267	

Seaman-Mannello	Q. Case disposition	.417	
	R. Personal/professional satisfaction	.682	**0.693** ^a^
	S. Inclination to identify	.585	**0.691** ^a^
	T. Perception of personal characteristics	.566	
	U. Personal attitudes toward drinking	.671	**0.775**

SAAPPQ	(1) Motivation	.426	
	(2) Work satisfaction	.725	**0.725**
	(3) Role adequacy	.848	**0.848**
	(4) Role legitimacy	.818	**0.818**
	(5) Task-specific self-esteem	.760	**0.760**

^a^Close to  .7, these subscales were included in order to capture a wide selection of attitudes.

**Table 3:** Attitude scores for the subscales of The Marcus Alcoholism Questionnaire, the Seaman-Mannello Nurses' Attitudes toward Alcohol and Alcoholism Scale, and the Shortened Alcohol and Alcohol Problems Perception Questionnaire.

Scale	Subscale	M	SD	Extreme negative responders (%)^a^
Marcus	Prognosis for recovery (recovery)	3.01	1.37	—
	Alcoholism and character defect (character defect)	2.59	1.22	—
	Social status of the alcoholic (social status)	2.91	1.02	—
	Alcoholism as an illness (illness)	3.13	1.67	4.2

Seaman-Mannello	Personal/professional satisfaction (satisfaction)	3.17	1.27	12.5
	Inclination to identify (“helpable”)	4.17	.982	—
	Personal attitudes toward drinking (personal drinking)	4.56	1.15	—

SAAPPQ	Work satisfaction (satisfaction)	3.66	1.11	8.2
	Role adequacy	4.04	1.33	4.1
	Role legitimacy	4.87	1.14	2.0
	Task specific self-esteem (self-esteem)	4.96	1.22	16.7
	I want to work with drinkers (desire)	3.40	1.23	14.3
	Pessimism is the most attitude to take toward drinkers (pessimism)	2.85	1.40	2.0

^a^For scales of recovery potential, character defect, social status, illness, and pessimism, a score of 7 represented an extreme negative response, while scales of satisfaction, “helpable,” personal drinking, work satisfaction, role adequacy, role legitimacy, self-esteem, and desire, a score of 1 indicated a strong negative response.

**Table 4:** Results of Multiple Regression Analyses of SAAPPQ subscales from Sociodemographic variables, The Marcus Alcoholism Questionnaire Subscales and the Seaman-Mannello Nurses' Attitudes toward Alcohol and Alcoholism Subscales. As outlined in Section 2, final models contain only significant individual predictors and/or variables that impacted on the significance or parameter estimates of others [44].

Subscale	Variable	*R* ^2^	*F* change	*β*	*t*
Work satisfaction	Satisfaction	0.416	32.748**	0.645	5.723**
Self-esteem	Consuming alcohol once a week	0.184		0.256	2.208*
	Recovery potential	0.454	18.705**	−0.547	−4.712**
Desire	Consume alcohol more than once a week			0.274	2.238*
	Social status			0.205	1.668
	Satisfaction	0.380	8.573**	0.524	4.264**
Pessimism	Consuming more than 2 standard drinks	0.115		0.369	2.661*
	Character defect			0.404	2.977*
	Age	0.340	6.507*	−0.226	−1.658

*Note.* Role adequacy and role legitimacy models were omitted (not significant).

*P < .05,  **P < .001.
